# Liver alterations and detection of SARS-CoV-2 RNA and proteins in COVID-19 autopsies

**DOI:** 10.1007/s11357-022-00700-6

**Published:** 2022-12-17

**Authors:** Adrián Pesti, Krisztina Danics, Tibor Glasz, Tibor Várkonyi, Tamás Barbai, Andrea Reszegi, Ilona Kovalszky, István Vályi-Nagy, Deján Dobi, Gábor Lotz, Zsuzsa Schaff, András Kiss

**Affiliations:** 1 Department of Pathology, Forensic and Insurance Medicine, Semmelweis University, Budapest, Hungary; 2 Department of Pathology and Experimental Cancer Research, Semmelweis University, Budapest, Hungary; 3 Central Hospital of Southern Pest - National Institute of Hematology and Infectious Diseases, Budapest, Hungary

**Keywords:** COVID-19, Endothelium, Immunohistochemistry, In situ hybridization, Liver, SARS-CoV-2

## Abstract

The most severe alterations in Coronavirus disease 2019 (COVID-19) caused by the severe acute respiratory syndrome Coronavirus-2 (SARS-CoV-2) infection are seen in the lung. However, other organs also are affected. Here, we report histopathologic findings in the liver and detection of viral proteins and RNA in COVID-19 autopsies performed at the Semmelweis University (Budapest, Hungary). Between March 2020 through March 2022, 150 autopsies on patients who died of COVID-19 were analyzed. Cause-of-death categories were formed based on the association with SARS-CoV-2 as strong, contributive, or weak. Samples for histopathologic study were obtained from all organs, fixed in formalin, and embedded in paraffin (FFPE). Immunohistochemical study (IHC) to detect SARS-CoV-2 spike protein and nucleocapsid protein (NP), CD31, claudin-5, factor VIII, macrosialin (CD68), and cytokeratin 7, with reverse transcriptase polymerase chain reaction (RT-PCR), and *in situ* hybridization (ISH, RNAscope®) for SARS-CoV-2 RNA were conducted using FFPE samples of livers taken from 20 autopsies performed ≤ 2 days postmortem. All glass slides were scanned; the digital images were evaluated by semiquantitative scoring and scores were analyzed statistically. Steatosis, single-cell and focal/zonal hepatocyte necrosis, portal fibrosis, and chronic inflammation were found in varying percentages. Sinusoidal ectasia, endothelial cell disruption, and fibrin-filled sinusoids were seen in all cases; these were assessed semiquantitatively for severity (SEF scored). SEF scores did not correlate with cause-of-death categories (*p* = 0.92) or with severity of lung alterations (*p* = 0.96). SARS-CoV-2 RNA was detected in 13/20 cases by PCR and in 9/20 by ISH, with IHC demonstration of spike protein in 4/20 cases and NP in 15/20. Viral RNA and proteins were located in endothelial and Kupffer cells, and in portal macrophages, but not in hepatocytes and cholangiocytes. In conclusion, endothelial damage (SEF scores) was the most common alteration in the liver and was a characteristic, but not specific alteration in COVID-19, suggesting an important role in the pathogenesis of COVID-19-associated liver disease. Detection of SARS-CoV-2 RNA and viral proteins in liver non-parenchymal cells suggests that while the most extended primary viral cytotoxic effect occurs in the lung, viral components are present in other organs too, as in the liver. The necrosis/apoptosis and endothelial damage associated with viral infection in COVID-19 suggest that those patients who survive more severe COVID-19 may face prolonged liver repair and accordingly should be followed regularly in the post-COVID period.

## Introduction

Since the outbreak of pandemic Coronavirus disease 2019 (COVID-19) caused by the severe acute respiratory syndrome Coronavirus-2 (SARS-CoV-2), alterations have been detected in several organs, based on data obtained at autopsies [1–28]. It is generally agreed that the most severe changes, closely associated with death of patients, occur in the lungs. However, other organs, including the liver, also are affected by SARS-CoV-2 infection [10, 29–35]. How specific alterations in different organs are is unclear, however, as is whether they represent a direct cytopathic effect of the virus or are caused by secondary factors such as hypoxia, cardiovascular disturbances, drug-induced injury, and cytokine storm [9, 10, 17, 32, 36, 37]. Nor are pathomechanisms [20, 38] and the extent of vascular injury in the liver during COVID-19 clear [39], including whether components of SARS-CoV-2 are present in parenchymal and non-parenchymal liver cells [30].

Several authors have highlighted the threat to health, especially the poorer outcomes of COVID-19 in older persons, that has been associated with aging of the immune system (immunosenescence and inflammageing), including diminished responses to viral infections, remodeling of immune responses, and increased “vulnerability” of the elderly [40–44]. Adults over 65 years of age and those with pre-existing chronic diseases (hypertension; diabetes; cardiovascular, renal, and liver diseases) are numerous among those dying of COVID-19, and aging is a critical and independent host factor in severity and progression of COVID-19 [9, 41, 45, 46]. Increased cellular senescence, when cells are not proliferating, being instead in permanent cell cycle arrest, might be caused by extrinsic and intrinsic factors, including viral infections, in addition to aging [47].

Some data suggest that chronic liver disease predisposes to more severe COVID-19 outcome and even to death, especially in combination with other co-morbidities. However, no detailed histological study has been conducted in this respect [29, 30, 48]. Thus, the main goal of our recent study, following the data published by our group on 100 COVID-19 autopsies, was to analyze liver histopathology in autopsy material, utilizing a classification of causes of death suggested previously [9]. We aimed to differentiate the specific and non-specific changes associated with COVID-19 in connection with the detection of SARS-CoV-2 spike protein and NP and viral RNA in situ in parenchymal and non-parenchymal liver cells.

## Patients and methods

Between March 2020 through March 2022, 150 autopsies on patients who died with COVID-19 were analyzed. The autopsies were performed using standard infectious-disease protocol in the Department of Pathology, Forensic and Insurance Medicine of the Semmelweis University, Budapest, Hungary, where about 3000 autopsies are conducted annually.

The diagnosis of COVID-19 was based on the results of ante-mortem reverse transcriptase polymerase chain reaction (RT-PCR) testing at the Department of Laboratory Medicine of Semmelweis University, which demonstrated SARS-CoV-2 sequences in nasopharyngeal swab elutes. The study was in accordance with the Declaration of Helsinki and was approved by the Central Medical Ethical Committee, Budapest, Hungary (No.IV/3961–2/2020/EKU, IV/4986–1/2020/EKU).

Demographic data (age, gender) of the patients and co-morbidities such as hypertension; diabetes; cardiovascular, cerebrovascular, respiratory, renal, and hepatobiliary diseases; malignant tumors; body mass index (BMI); and length of hospital stay are given in Table 1. No data on previous liver biopsy samples taken from the 10 deceased with chronic liver disease were available, after checking the institutional central database based on their national social security identification numbers (TAJs).

**Table 1:** Demographic and clinical data of 150 patients infected with SARS-CoV-2 and analyzed at autopsies

*n* (number of patients)	150
Females; *n* (%)	69 (46.0)
Age, years; mean (SD)	69.61 (14.31)
Age, females, years; mean (SD)	72.88 (13.57)
Age, males, years; mean (SD)	66.81 (14.41)
Age categories, years; *n* (%)	
< 51	17 (11.33)
51–60	18 (12.0)
61–70	39 (26.0)
71–80	41 (27.33)
81–90	26 (17.33)
> 90	9 (6.0)
Co-morbidities in patient history; *n* (%)	
Hypertension	106 (70.67)
Cardiovascular diseases	82 (54.67)
Diabetes	50 (33.33)
Cerebrovascular diseases	41 (27.33)
Respiratory diseases	37 (24.67)
Malignant tumors	25 (16.67)
Renal diseases	24 (16.00)
Diseases of the central nervous system	20 (13.33)
Liver diseases	10 (6.67)
BMI, kg/m^2^; mean (SD)	27.98 (6.70)
BMI categories; *n* (%)	
< 18.5	3 (2.0)
18.5–24.9	58 (38.67)
25–29.9	41 (27.33)
> 30	48 (32.0)
Length of hospital stay, days; mean (SD)	17.26 (16.66)
Length of hospital stay categories, days; *n* (%)	
< 1	8 (5.33)
1–2	18 (12.0)
3–5	11 (7.33)
6–9	20 (13.33)
10–15	28 (18.67)
> 15	65 (43.3)
Intensive care = yes; *n* (%)	83 (55.33)
Cause-of-death categories; *n* (%)	
Strong COVID-19	79 (52.67)
Contributive COVID-19	47 (31.33)
Weak COVID-19	24 (16.0)

*SD*, standard deviation; *BMI*, body mass index

**Fig. 1:**
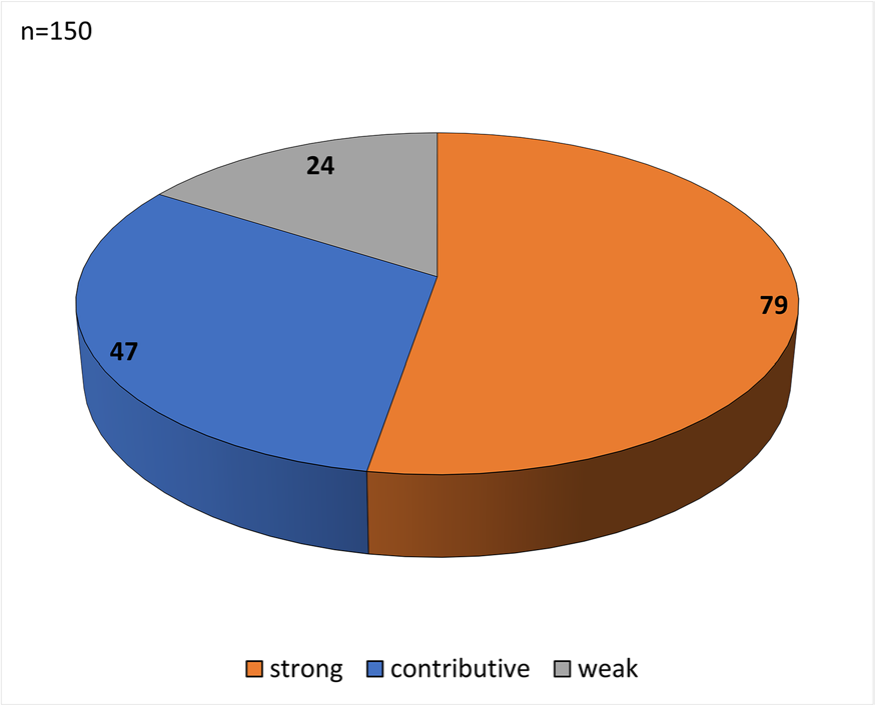
Cause-of-death categories of 150 deceased based on association with SARS-CoV-2 as strong, contributive, and weak

One of three cause-of-death categories was assigned to each patient, based on the relevance of SARS-CoV-2 infection to the cause of death. These reflected (1) strong, (2) contributive, and (3) weak associations, as defined previously by our group (Fig. 1) [9].

Selected laboratory data such as values for serum activities of aspartate aminotransferase (AST), alanine aminotransferase (ALT), alkaline phosphatase (ALP), and gamma-glutamyl transferase (GGT), serum bilirubin and C-reactive protein concentrations, and lymphocyte and neutrophil granulocyte counts are presented in Table 2. The median values of biochemical data obtained during the 6 to 7 days before patient death were recorded as described [9].

**Table 2:** Selected laboratory data of 150 autopsied patients infected with SARS-CoV-2

Total number of patients *n* = 150	Number of patients with available laboratory values	Number of patients with abnormal laboratory values (%)
Elevated AST activity Normal range: 13–40 U/l	120	57 (47.5)
Elevated ALT activity Normal range: 7–40 U/l	120	51 (42.5)
Elevated ALP activity Normal range: 40–130 U/l	120	46 (38.3)
Elevated GGT activity Normal range: 12–52 U/l	120	91 (75.8)
Elevated bilirubin concentration Normal range: 5–21 µmol/l	119	29 (24.4)
Elevated CRP concentration Normal range: < 10 mg/l	129	124 (96.1)
Low lymphocyte count Normal range: 1.5–4 G/l	131	98 (74.8)
Elevated neutrophil count Normal range: 2–7.5 G/l	131	88 (67.2)

The postmortem interval (PMI) before autopsy varied between 0.25 and 17 days (average 3.5 days). For detection of SARS-CoV-2 viral proteins, viral genomic RNA, and endothelial antigens, selected samples taken from cases with PMI ≤ 2 days were used (20 “rapid autopsies”). Histopathological changes such as steatosis, fibrosis, and inflammation, in the liver were studied in all samples, regardless of PMI. Histopathologic findings in the lung were evaluated and scored as well. Total pulmonary scores (TP scores) were created as before to express the severity of the alterations (*v.i.*) [9].

### Gross anatomy and histology

**Fig. 2:**
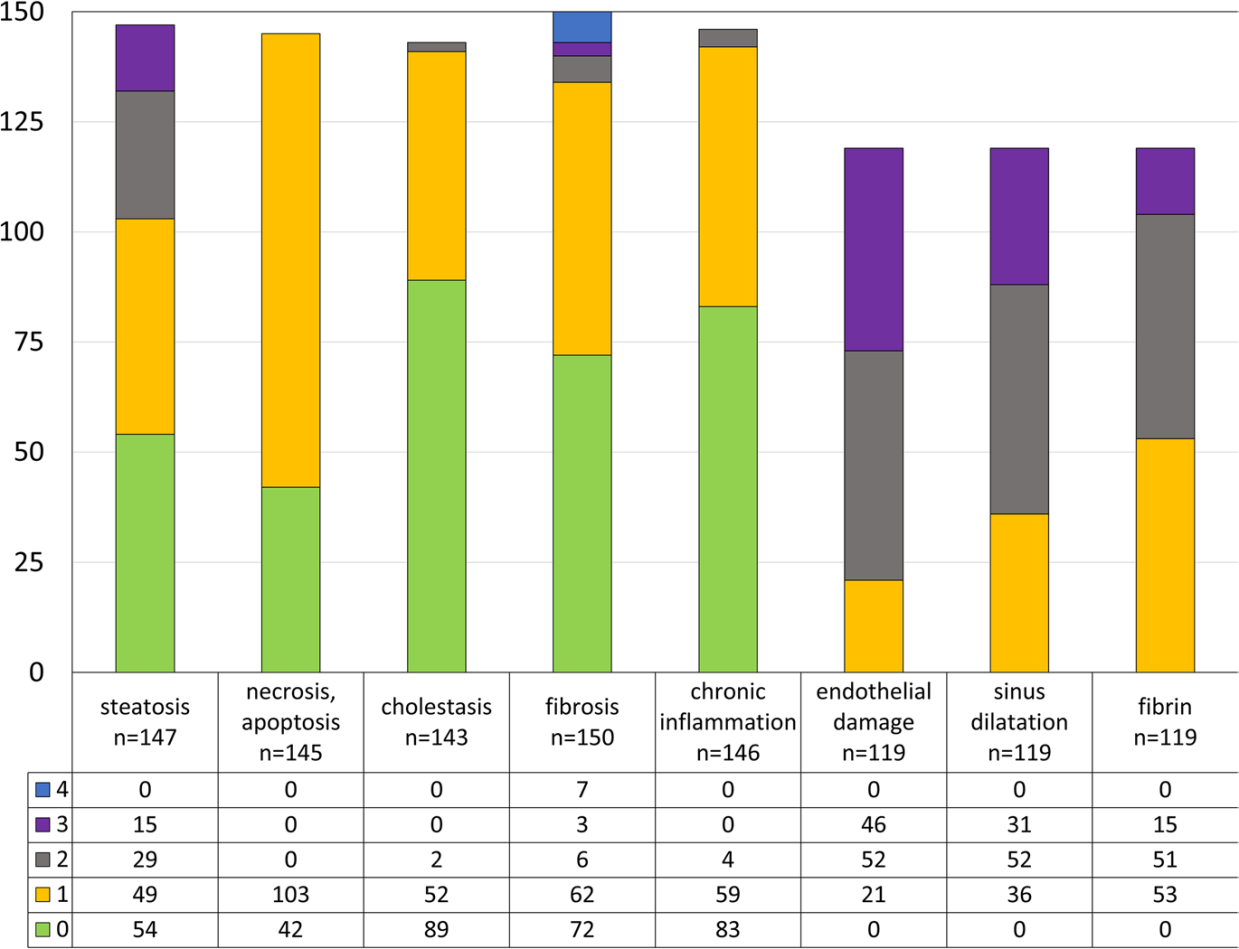
Severity of the main histological alterations in the liver of patients died of COVID-19. Scores: 0–3, except fibrosis: 0–4

The organs were weighed. The liver was sampled at 4–5 sites for histopathologic study. Samples also were taken from other organs (lungs, heart, kidneys, oropharynx, thyroid glands, spleen, adrenal glands, gastrointestinal tract, brain) and from sites macroscopically altered. The tissue blocks were fixed in 10% buffered formalin with a final formaldehyde concentration of 4%, embedded in paraffin (FFPE). Sections were stained with hematoxylin and eosin (HE) and periodic acid-Schiff techniques without (PAS) and with (dPAS) amylase digestion. All HE-stained slides were scanned using a 3DHistech Pannoramic® 1000 Digital Slide Scanner (3DHistech Ltd, Budapest, Hungary) with 82 × optical magnification (0.121 µm/pixel resolution). The severity of the alterations in the lung, heart, and kidney was evaluated by 2 board-certified pathologists and that in the liver by 3 hepatopathology specialists (Zs. S., A. K., G. L.). Evaluations were conducted independently, and questionable images were discussed together to achieve consensus as described [9]. Severity of the alterations in the liver was analyzed semiquantitatively (Fig. 2) in respect of steatosis, cholestasis, chronic inflammation, sinusoidal ectasia, endothelial damage, and fibrin-filled sinusoids: 0 = none, 1 = mild, 2 = moderate, 3 = severe; single-cell apoptosis or necrosis: 0 = none, 1 = 1–5%, 2 = 6–20%, 3 ≥ 20%; fibrosis: F0–F4. Based on scored extents of sinusoidal ectasia (*S*), endothelial damage (*E*), and fibrin-filled sinusoids (*F*), SEF scores were created for individual cases by adding the three scored values. Correlation then was sought among SEF scores, severity of lung alterations, and cause-of-death categories. Severity of the pulmonary alterations was analyzed as described, applying the total pulmonary score (TP score) as statistical comparison [9]. Alterations scored in the lung included thrombosis/embolus, fibrin macrothrombus/microthrombus, diffuse alveolar damage (exudative, proliferative, and fibrotic phases), hemorrhagic infarction, alveolar edema, and leukocyte reaction.

### Immunohistochemistry

Immunohistochemical (IHC) analyses were conducted using sections of FFPE liver blocks cut at 3–5 µm and picked up on coated glass slides. IHC reactions were carried out in 20 cases in which the PMI was ≤ 2 days. To detect SARS-CoV-2 NP, a mouse monoclonal antibody (IgG1, clone #05, Cat. No. 40143-MM05, Sino Biological, Beijing, People’s Republic of China) in 1:1000 dilution was used and to detect SARS-CoV-2 spike (S1) protein a rabbit monoclonal antibody (IgG1, clone E5S3V, Cat. No. 99423, Cell Signaling Technology, Danvers, MA) in 1:500 dilution was used. The sensitivity and specificity of different anti-SARS-CoV-2 antibodies were checked and selected in a nationwide study [49]. Further antibodies against CD31, CD34, and claudin-5 for endothelial cells, factor VIII for fibrin, CD68 for macrophages, and CK7 for cholangiocytes, with species-specific secondary antibodies, were used as listed in Table 3. Stainings were carried out in a Ventana Benchmark Ultra automated IHC system (Ventana Medical Systems, Tucson, AZ) according to assay instructions, using CC1 antigen retrieval (pH 8.0), signal developed with OptiView DAB diaminobenzidine IHC Detection Kit (Ventana Medical Systems). The slides were counterstained with hematoxylin II (Ventana Medical Systems), dehydrated and mounted. For detection of SARS-CoV-2 spike protein and NP, UltraView Universal Alkaline Phosphatase Red Detection Kit (Ventana Medical Systems) was additionally used. The glass slides were scanned as described above.

**Table 3:** List of antibodies used in the study

Antigen	Host species (clone)	Manufacturer*	Cat. no	Dilution for IHC**
SARS-CoV-2 nucleoprotein	Mouse monoclonal (05)	Sino Biological	40143-MM05	1:1000
SARS-CoV-2 spike protein (S1)	Rabbit monoclonal (E5S3V)	Cell Signaling Technology	99423	1:500
CK7	Mouse monoclonal (OV-TL 12/30)	Dako	M7018	1:3500
CD31	Mouse monoclonal (JC70A)	Dako	M0823	1:300
CD34	Mouse monoclonal (QBEnd/10)	Leica	NCL-L-END	1:300
CD68	Mouse monoclonal (KP1)	Dako	M0814	1:4000
Claudin-5	Rabbit monoclonal (EPR7583)	Abcam	ab131259	1:200
Factor VIII	Mouse monoclonal (F8/86)	Dako	M0616	1:100

^*^Abcam, Cambridge, UK; Cell Signaling Technology, Danvers, MA; Dako, Glostrup, Denmark; Leica, Wetzlar, Germany; Sino Biological, Beijing, People’s Republic of China

^**^IHC, immunohistochemistry

### SARS-CoV-2 detection by RT-PCR assay

Total RNA was isolated from FFPE tissue sections using the RNeasy FFPE Kit (Qiagen®, Venlo, the Netherlands) according to the manufacturer's instructions. RNA yields and quality were determined using a NanoDrop 2000 spectrophotometer (Life Technologies of Thermo Fisher Scientific, Waltham, MA). SARS-CoV-2 Research Use Only qPCR Primer & Probe Kit (Cat. No. 10006770, Integrated DNA Technologies, Coralville, IA) contains premixed primers and probes for N1, N2, and RP detection used in individual reactions by RT-PCR. Assays for nCoV were performed on a Light Cycler 480 II system (Roche Diagnostics, Indianapolis, IN) with TaqPathTM 1-Step RT-qPCR Master Mix, CG (Cat. No. A15300, Thermo Fisher Scientific), according to the protocol of Centers for Disease Control and Prevention, Respiratory Viruses Branch, Division of Viral Diseases (Cat. No. 2019-nCoVEUA-01, Atlanta, GA).

### SARS-CoV-2 genomic RNA detection by in situ hybridization

*In situ* hybridization was performed for viral RNA detection in FFPE samples taken from 20 rapid autopsies, using the RNAscope® 2.5 HD Detection Reagents-RED (Cat. No. 322350, Advanced Cell Diagnostics—ACD, Bio-Techne, Newark, CA) and SARS-CoV-2 RNAscope® ISH Probe (bp 21631–23303 Cat. No. 848561, ACD) according to the manufacturer's protocol. In brief, deparaffinized sections were subjected to target retrieval for 20 min at 98–102 °C in 1 × Target Retrieval Solution and to Protease Plus treatment for 15 min at 40 °C in a HybEZ™ oven (ACD). 50% Gill hematoxylin I (Sigma Aldrich, St Louis, MO) staining solution was used for counterstaining for 2 min at room temperature. SARS-CoV-2 positive lung and placenta tissues were used as positive tissue controls and additional liver samples were used as negative and positive probe controls to check assay specificity. Digitalized images of sections were generated as described above.

### Statistical analysis

All analyses were performed using Microsoft Office 365 Excel, version 2207 (Microsoft Corporation, Redmond, WA) and IBM SPSS Statistics, version 28.0.1.0 (IBM Corporation, Armonk, NY). Continuous data are presented as the mean ± standard deviation (SD) and categorical data as numbers and percentages. Associations between groups and categories were analyzed by one-way ANOVA testing with appropriate post hoc tests. Normal distribution assumptions were analyzed by histograms. *P*-value < 0.05 was considered statistically significant for all analyses.

## Results

### Demographic and clinical data

The mean age of the autopsy cohort was 69.6 years, with 69 females and 81 males. Most patients had at least one co-morbidity, with hypertension, cardiovascular disease, and diabetes the most common. The BMI was between 25 and 29.9 in 27.33% and > 30 in 32% of patients. The length of hospital stay was 17.26 days (from < 1 to > 15); 83 patients (55%) were treated in intensive care units (Table 1). The causes of death in the cohort were categorized as strongly (79 cases), contributively (47 cases), or weakly (24 cases) associated with SARS-CoV-2 infection (Fig. 1).

### Histologic study with IHC

**Fig. 3:**
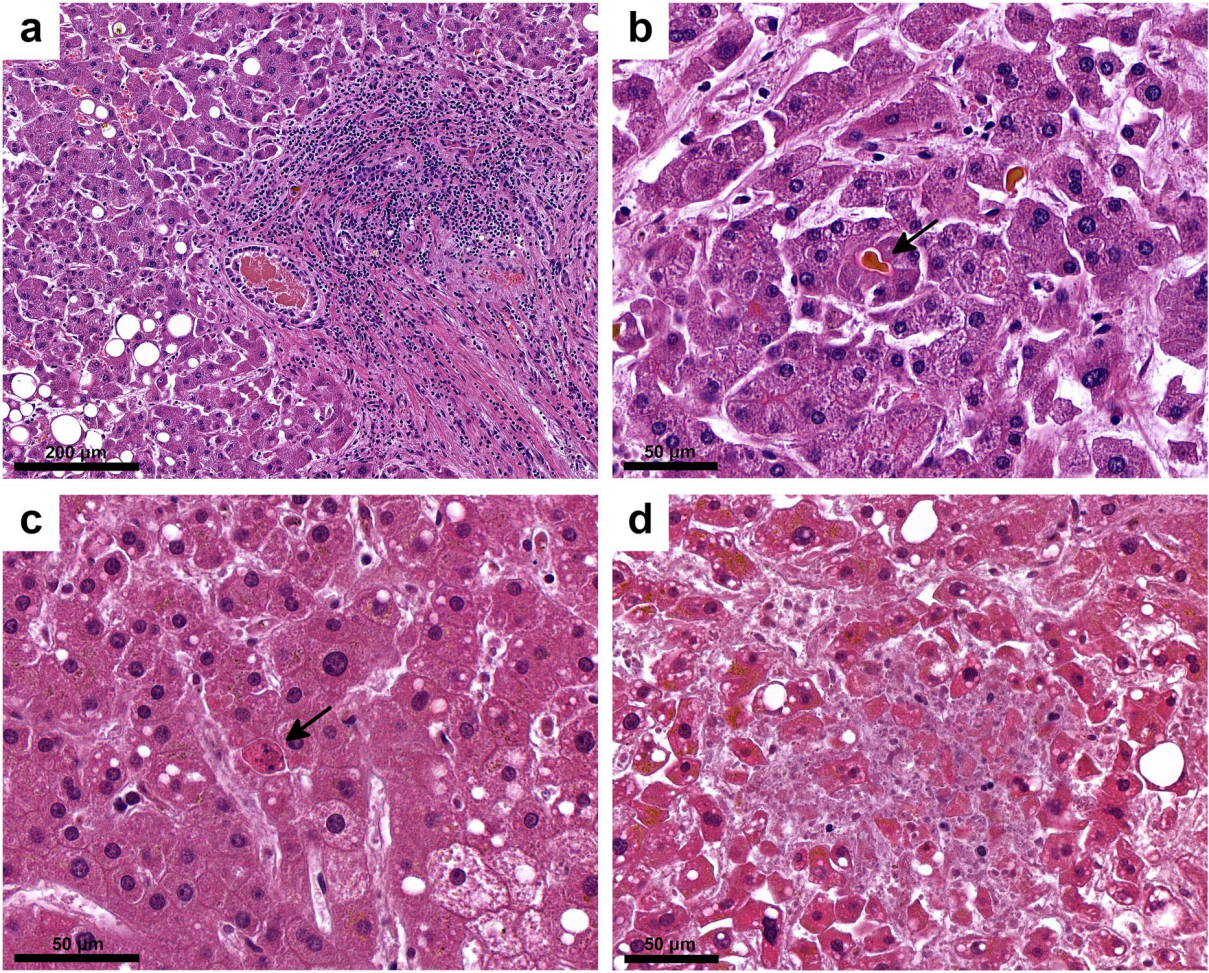
Steatosis, mild portal fibrosis and inflammatory infiltration (**a**), canalicular cholestasis (**b,** arrow), apoptotic body (**c**, arrow), and focal necrosis (**d**) in the liver of patients died of COVID-19. Hematoxylin/eosin (HE)

Figure 2 presents the main histopathologic alterations detected in COVID-19 livers. A few cases could not be evaluated because of advanced autolysis (especially affecting endothelium). Hepatic lobular architecture was preserved in most cases, excepting 7 with cirrhosis (F4; 4.67%). 3/150 were F3 (2%), 68/150 F1-2 (45.33%), while no fibrosis (F0) was seen in 72/150 cases (48%). Hepatocyte steatosis was present in a high proportion of cases (93/147, 63.27%), in varying degrees (Fig. 3a). No significant lobular, portal, or interface inflammatory infiltration was detected in most of the cases (Figs. 2 and 3a). The grade of inflammation, where present, was mild (grade 3/18 histology activity scores). An increase in CD68-expressing macrophages was detected, especially in zone 3 (not shown). Most bile ducts (identified by CK7 expression) were normal, but ductular reaction was detected in cases with cirrhosis and intralobular canalicular cholestasis was seen in 54 of 143 cases (Fig. 3b). Single-cell necrosis/apoptosis of hepatocytes (Fig. 3c) and focal or centrilobular zonal necrosis, when seen, lacked all lymphocyte or neutrophil-leukocyte inflammatory reaction (Fig. 3d).

**Fig. 4:**
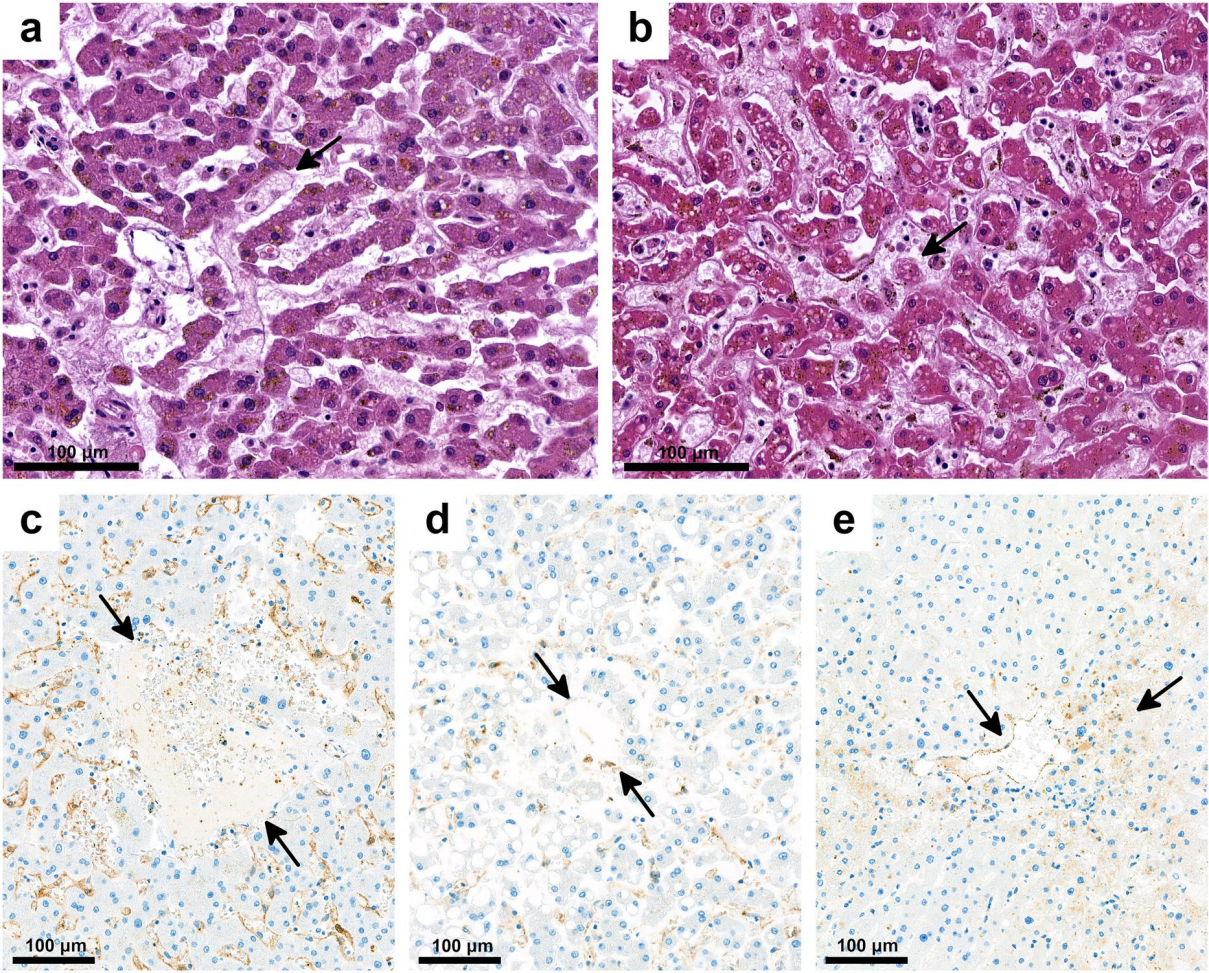
Vascular and sinusoidal endothelial damage in the liver in COVID-19. Damaged endothelial lining, extension of Disse space, “ghost” red blood cells (arrow) (**a**), ectatic sinusoids with fibrin and cellular debris (**b**, arrow). Immunohistochemistry with claudin-5 (**c**), CD31 highlights the loss of endothelial staining (**d**, arrows). The endothelial leakage is seen by FactorVIII reaction (**e**, arrows). (**a**, **b** HE; **c** IHC with CD31; **d** IHC with caludin-5; **d** IHC with FactorVIII, IHC, immunohistochemical reaction)

**Fig. 5:**
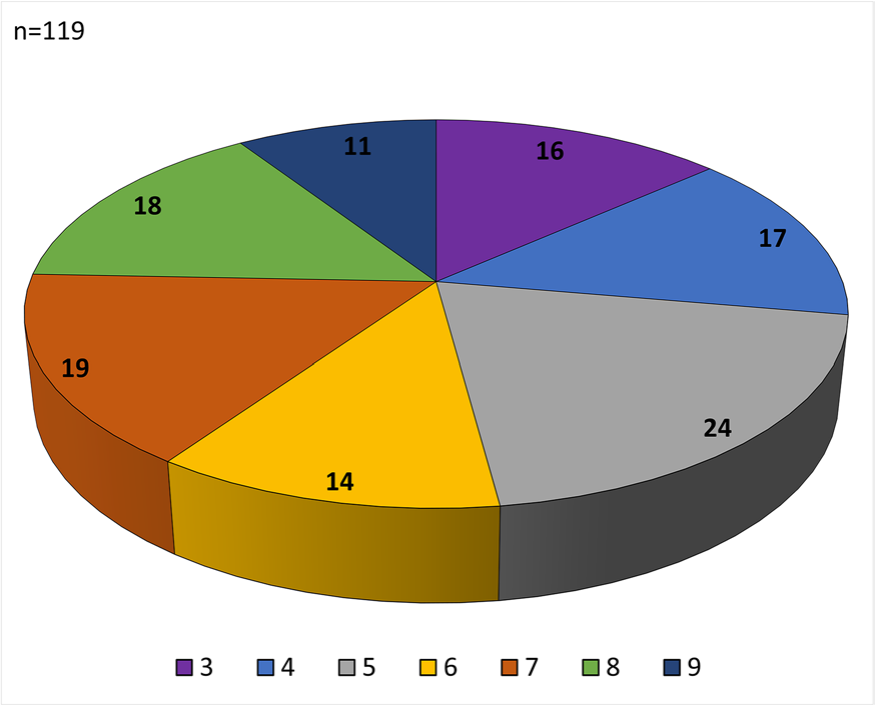
Distribution of the severity of sinusoidal alterations expressed as SEF scores in the liver of 119 patients died of COVID-19. The scores were created by adding the scores for each case of sinusoidal ectasia (*S*, scores 0–3), endothelial damage (*E*, scores 0–3), and fibrin (*F*, scores 0–3). The maximal scores for each case were 9 scores

In contrast to the mild alterations seen in parenchymal liver cells, severe changes were detected in the vascular and sinusoidal endothelial cells in all cases suitable for analysis (119/150). Endothelial disruption and endothelial cell destruction were found (Figs. 4a, b and 2), highlighted by claudin-5 and CD31 IHC (Figs. 4c, d). Leakage of endothelial lining was demonstrated by factor VIII IHC (Fig. 4e). The changes were associated with ectasia of sinusoidal lumina and of the space of Disse. Zone 3 areas were flooded by homogenous, occasionally fibrillary, light blue fibrin (Fig. 4a, b), similar to the material detected in fibrin thrombi or exudate in lung alveolar spaces. Semiquantitative evaluation by SEF scoring was conducted for each individual case by adding the scores for sinusoidal ectasia (*S*, scores 0–3), endothelial damage (*E*, scores 0–3), and fibrin accumulation within sinusoids (*F*, scores 0–3), with 9 the highest possible SEF score. Figure 5 presents the severity of the alterations in SEF score terms, with scores > 6 in most cases. Occasional platelet–fibrin microthrombi were seen.

The average age of the 150 patients were 69.6 years (26–101 years), those who’s samples were analyzed for SEF were 69.4 years (26–101). There was no significant difference in the severity of alterations between younger (< 65) and older (≥ 65) age groups, comparing the total SEF scores. Furthermore, we analyzed the severity (grade) of different alterations individually, as sinusoidal dilatation, endothelial damage, and fibrin and compared with the age of the decease; however, no statistical difference was found (*p* = 0.289 for sinusoidal dilatation, *p* = 0.606 for endothelial damage, *p* = 0.054 for fibrin).

### Correlation between liver and lung alterations and cause-of-death categories

TP scores were not correlated with severity of necrosis/apoptosis (*p* = 0.87) or with steatosis (*p* = 0.44) in the liver. SEF scores were not correlated with TP scores (*p* = 0.96) or with cause-of-death categories (*p* = 0.92).

### Detection of SARS-CoV2 RNA by PCR and in situ hybridization; detection of SARS-CoV2 proteins by immunohistochemistry

Among the 20 individuals who underwent rapid autopsy, 18 lung and 13 liver samples contained SARS-CoV-2 RNA demonstrable by RT-PCR (Table 4). One of the patients in whom SARS-CoV-2 RNA was not found in lung using RT-PCR had demonstrable SARS-CoV-2 RNA in liver (#18) and the other did not (#7).

**Table 4:**
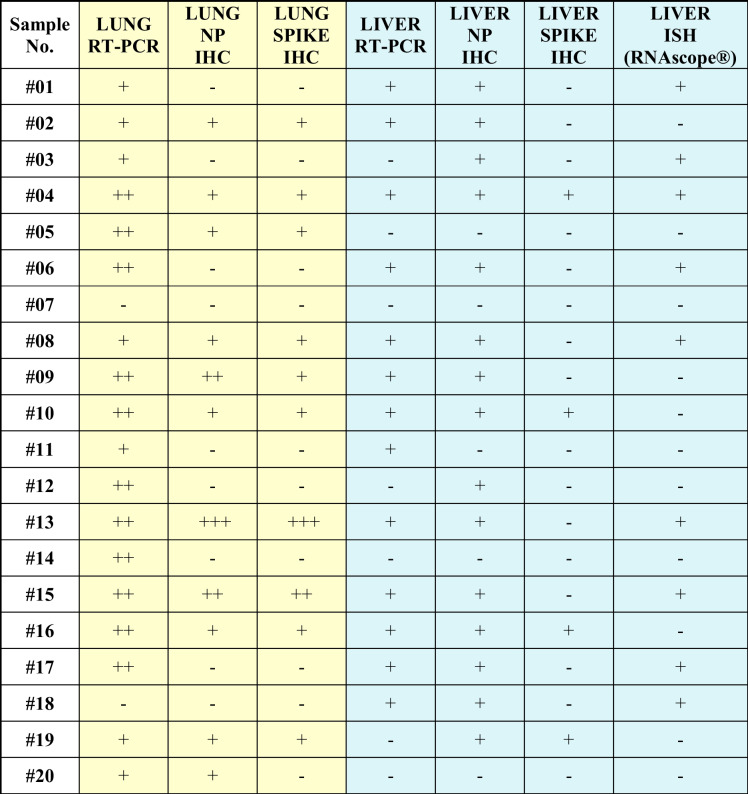
Detection of SARS-CoV-2 RNA by RT-PCR, ISH, and of spike protein and NP by IHC in livers from autopsies performed ≤ 2 days postmortem

*RT-PCR*, reverse transcriptase polymerase chain reaction; *NP*, nucleoprotein; *IHC*, immunohistochemistry; ISH, *in situ* hybridization

Viral RNA sequences were detected by ISH in 9/20 liver samples, with parallel positivity by PCR in the same patients’ livers (Table 4). In 5 cases with PCR-demonstrable viral RNA, however, no viral RNA was detected by ISH (#2, #9, #10, #11, #16).

**Fig. 6:**
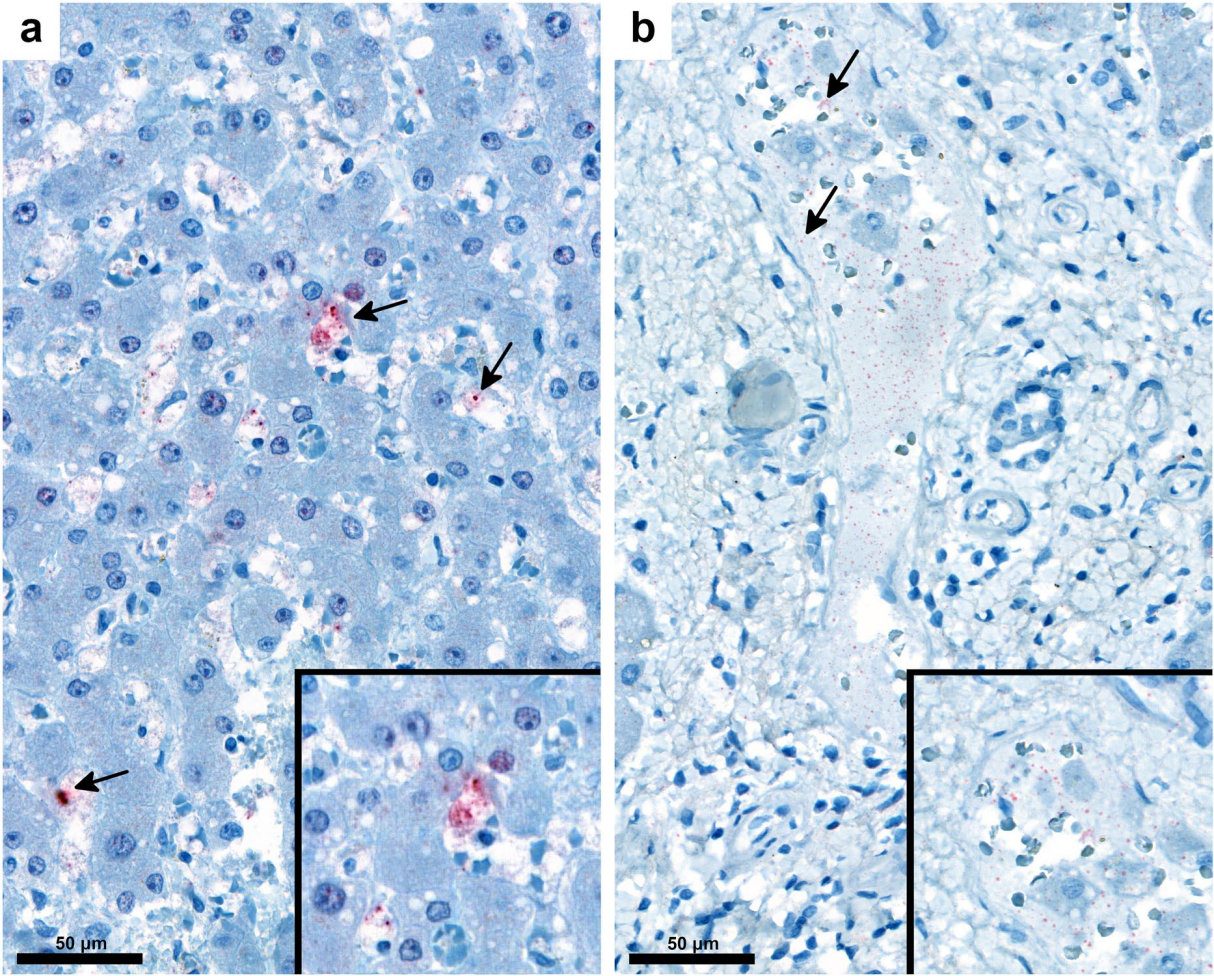
Detection of SARS-CoV-2 nucleocapsid (**a**, arrows) and spike proteins (**b**, arrows) are located in vascular and sinusoidal spaces, in endothelial and Kupffer cells by IHC. Inserts show positive reactions with higher magnification (180 ×). (**a**, **b**, inserts: IHC, alkaline phosphatase red, IHC, immunohistochemical reaction)

**Fig. 7:**
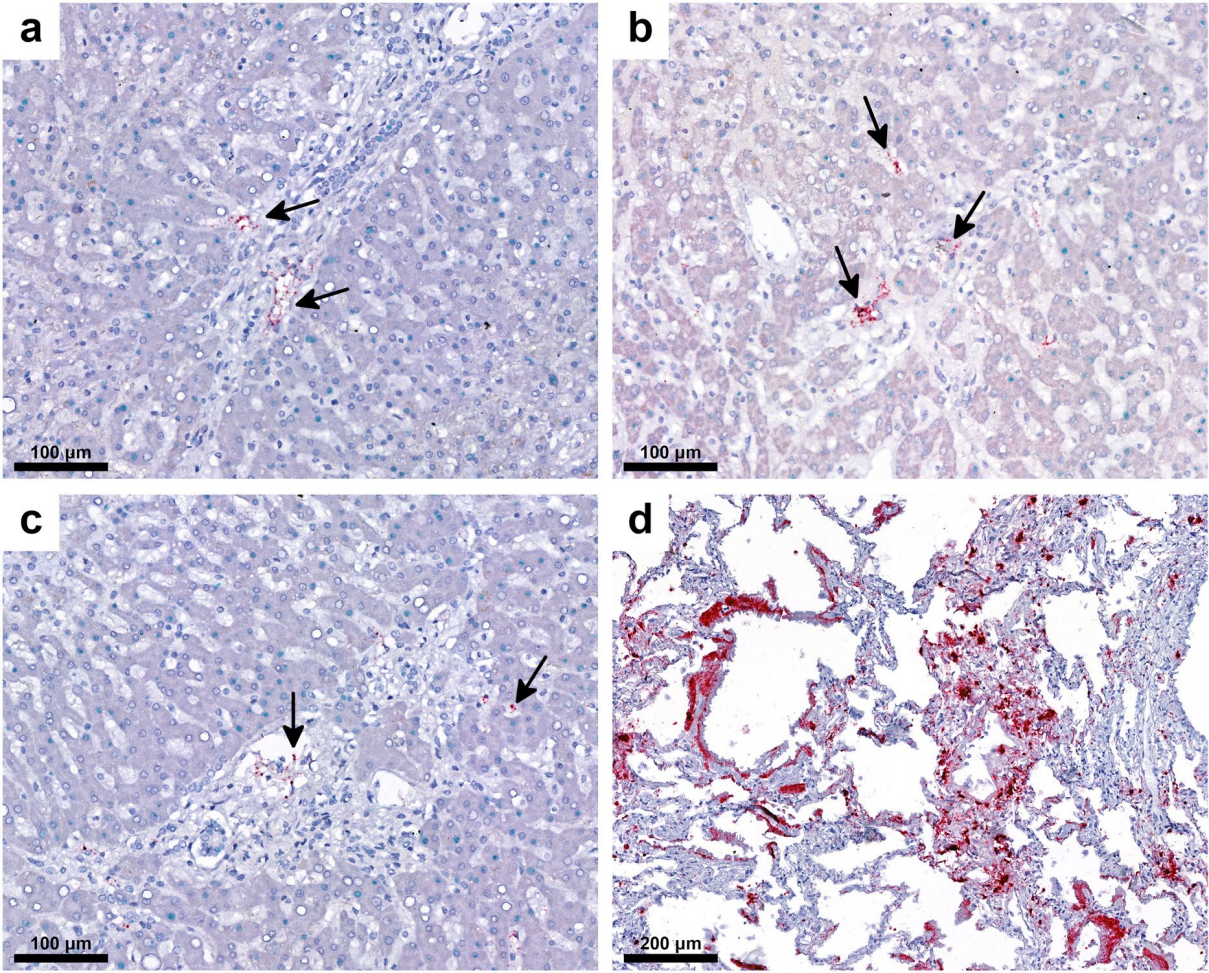
Detection of SARS-CoV-2 RNA by *in situ* hybridization. The red signals can be seen in endothelial, Kupffer cells, and portal macrophages (**a**, **b**, **c**, arrows). Strong red reaction signals in lung tissue infected with SARS-CoV-2 as positive control (**d**). RNAscope®

IHC was used to detect SARS-CoV-2 NP (Fig. 6a) and spike protein (Fig. 6b). Viral NP and spike protein were unevenly and focally distributed in endothelial and Kupffer cells and in portal macrophages but were not found in hepatocytes or cholangiocytes (Fig. 6a, b). Signals of viral RNA by ISH and proteins by IHC were detected in similar localizations and distributions (Fig. 7).

SARS-CoV-2 RNA was demonstrated by PCR in parallel with NP demonstrated by IHC in 12/20 cases. Three cases contained IHC-demonstrable NP whilst RNA was not found by PCR (Table 4). The spike protein was demonstrated in 4 cases only (#4, #10, #16, #19), of which 3 contained PCR-demonstrable RNA (#4, #10, #16) and one ISH-demonstrable RNA (#4). Both NP and spike protein were demonstrated in 4 cases. No spike protein expression was found by IHC without expression of NP.

Viral components were detected in 15 cases out of the 20 cases, while no virus in 5 out of them by IHC and/or ISH in the liver. In 4 out of 5 cases, the liver RT-PCR was negative as well (Table 4). We compared the severity of the sinusoidal dilatation, endothelial damage, and fibrin (scores) of the 5 virus-component negative and 15 virus-component positive cases; however, no significant differences were detected (*p* = 1 for sinusoidal dilatation, *p* = 0.234 for endothelial damage, *p* = 0.853 for fibrin).

### Laboratory data

Elevated AST activity was observed in 47.5% of patients, ALT in 42.5%, ALP in 38.3%, and GGT in 75.8%. CRP was elevated in 96.1% of patients, and bilirubin in 24.4%. The lymphocyte count was low in 74.8% of cases and the neutrophil-leukocyte count was high in 67.2% of cases. No correlation was seen in any biochemistry data and liver histology alterations as steatosis, necrosis/apoptosis, cholestasis, fibrosis, chronic inflammation, and SEF scores (Table 2).

## Discussion

Several factors associated with aging are thought to worsen outcome in COVID-19. These include accumulation of DNA damage, decreased autophagic activity, telomerase shortening, cellular and metabolic stress, and immunosenescence, in combination leading to increased numbers of senescent cells [50]. The “irreversible loss of cell proliferation and cell cycle growth arrest” that define cellular senescence results in increased production of chemo- and cytokines, termed “cytokine storm,” detected often in the older [47, 50]. Morphological, metabolic, and molecular alterations characterize the senescent cells, with a senescence-associated secretory phenotype that includes increased production of inflammatory cytokines (e.g*.*, interleukins 6 and 1α, HMGB-1), pro-coagulatory mediators, and extracellular matrix-active factors [51]. Severe cellular and tissue damage occurs not only in the lung, as the primary site of infection, but also in other organs, including the liver. Whether cellular injury is a direct viral cytopathic effect, is induced by cytokine storm, or results from treatment is still an open question, and the cells involved in viral replication in the liver in COVID-19 are not definitively identified.

Several studies have analyzed the changes in the livers of those dying with COVID-19 [52]. Steatosis was found in high percentages, together with mild portal inflammation, portal fibrosis, and hepatocyte necrosis and apoptosis, in our study as well in others [9, 13, 17, 30, 32, 36, 53]. A meta-analysis estimated steatosis of varying degree in 5.1% of cases, portal inflammation in 13.3%, fibrosis in 20.5%, and lobular inflammation in 11.6% [10]. Our previous [9] and recent findings in 150 autopsy cases included steatosis in 63% and fibrosis in 52%. There was no correlation among TP scores (*p* = 0.44), cause-of-death categories (*p* = 0.13), and steatosis. This may suggest that steatosis, commonly seen in COVID-19 livers, is not associated directly with the severity of SARS-CoV-2 infection—at least not with its severity as manifest in the lungs of the deceased. Steatosis might thus be interpreted as a secondary change associated with co-morbidities or treatments rather than as a primary viral cytotoxic effect.

Liver fibrosis and inflammatory reaction were mild in most cases, as others have found [13]. This suggests that fibrosis, detected in a certain percentage of cases, is not the consequence of COVID-19, at least not in the acute phase of infection, but is rather associated with previous diseases. Only 7 members of our autopsy cohort had cirrhosis, and in their livers, we saw no severe acute alterations such as extended necrosis, hemorrhage, and inflammation; only structural nodularization and fibrosis of long standing. This may suggest that in its acute phase COVID-19 does not severely aggravate pre-existing chronic liver disease manifest as steatosis and fibrosis. Chronic liver disease seems as well not significantly to contribute to death of patients during the acute phase of COVID-19. Some reports, however, found that chronic liver diseases, especially cirrhosis, might predispose to more severe outcome in COVID-19 [30]. In those patients with chronic liver diseases who survive COVID-19, however, vascular alterations like those demonstrated at autopsy might adversely affect evolution of chronic liver disease.

The common findings of single-cell necrosis/apoptosis and zonal necrosis detected in the liver in COVID-19 by several authors, us among them (103/145; 71%) strongly suggest that SARS-CoV-2 infection affects the liver, whether primarily or secondarily [5, 9, 39]. That viral RNA and proteins were not detected in hepatocytes and cholangiocytes suggests that the necrosis/apoptosis seen in COVID-19 livers marks not viral cytopathic effects but instead extrahepatic influences such as hypoxia, sepsis, circulatory disturbances, and treatments.

Altered biomarker values were regularly observed in COVID-19. In serum, elevated enzyme activities (ALT, AST, GGT), concentrations of bilirubin, and concentrations of the inflammation indicator CRP were found; low lymphocyte counts, and high neutrophil-leukocyte counts were detected as well [9, 54–58]. There was, however, no correlation found earlier between altered laboratory data and hepatopathologic findings in 27 and 100 autopsy cases [9, 33], and which is the same in the recent study. Dyshemostasis characterized COVID-19, with increased prothrombin time, low platelet count, and high levels of interleukin 6, fibrinogens, and von Willebrand factor, among other abnormalities [52].

Vascular endothelial dysregulation and damage, affecting especially sinusoidal endothelium, were usually very severe in our studies and in some others [9, 39, 59]. However, others found none [17] or found it in 20% of cases only [13]. Our study found no correlation among TP scores (*p* = 0.96), cause-of-death categories (*p* = 0.92), and SEF scores. CD31 and tight junction protein claudin-5 IHC permitted good visualization of the damaged and disrupted endothelial cells of the draining veins and sinusoidal lining. This was associated with abnormal ectasia of sinusoidal lumina, which were filled with fibrin, red blood cells and “empty” red blood cells (ghost cells), mainly in zone 3, permitting the inference that sinusoidal circulation of blood was altered. Similar changes were detected by others too [60]. Exsudative leakage could be well demonstrated by detection of factor VIII in zone 3 ectatic sinusoids. Based on these observations, SEF scoring was undertaken, and its results were compared with TP scores; however, the two were not correlated.

Our study detected SARS-CoV-2 spike protein and NP as well as viral RNA in the liver as in those by others [8, 17, 30, 31, 37, 61], albeit in low amounts and uneven distribution, in contrast to the high expression in lung tissue. Others did not observe SARS-CoV-2 staining in hepatocytes [33], and other liver cells [53, 62]. These findings accord with the data of others, who analyzed the distribution, quantification, and replication of SARS-CoV-2 in several organs in autopsies and found that N1 and N2 gene copies/ng RNA were low in the livers and varied from patients to patients (N1: 0.0079, 1.4177, N2: 0.0124, 0.0057, 0.0069, 1.5670 copies/ng RNA) [8]. In regard to the concordance rate (CR) of the different detection methods, RT-PCR for SARS-CoV-2 RNA and IHC for NP showed remarkably high CR (80%) considering that processes for gene regulation at the transcriptional (mRNA) and posttranscriptional (protein) levels are different. The low detection rate of the spike protein relative to the previous two may represent actual difference in expression levels between the two proteins, a phenomenon that has been reported previously [63].

The discrepancies among the detection rate might be explained by the different sensitivity of the methods applied on FFPE material, focal distribution of the viral components, and low expression of SARS-CoV-2 in the livers compared to the lungs. The uneven distribution of the infectious agent is a well-known factor in the limitations of tissue detection of other pathogens; the lower the pathogen load, the less uniform and patchy the staining pattern, either by IHC or ISH [64]. In extreme cases, this may result in the absence of the pathogen in one section plane by any tissue-based staining method. SARS-CoV-2 viral load in the liver is particularly low, it is therefore possible that no positive cells are present in the tissue sections prepared for ISH. However, for the SARS-CoV-2 RT-PCR assay, nucleic acid isolation was performed from multiple tissue sections, so there was a higher chance of finding virus-positive tissue fragments. This may also explain why some authors could not locate viral components in the liver [5, 13, 62]. Others emphasize, however, that the detection of SARS-CoV-2 genomic RNA is not necessary a sign of viral replication [65] and cells positive with ISH represent “either infection or phagocytized virus in resident macrophages” [8]. Differences were noted in the lung from COVID-19 autopsies as well in the detection sensitivity of tissues-based SARS-CoV-2 Assays comparing RT-PCR, whole-genome sequencing and ISH [66].

Endothelial cells express angiotensin-converting enzyme 2 (ACE2) and transmembrane protease serine subtype 2 (TMPRSS2) receptors, entry points for SARS-CoV-2 in pulmonary epithelial cells and several other cell types. However, the co-receptors for SARS-CoV-2 entry may not be the same in all organs [59]. Whether SARS-CoV-2 replicates in endothelial cells is still debated [59]. Recent studies detected replicating SARS-CoV-2 in endothelial cells of several organs [8, 31], permitting the suggestion that this may have a role in cytopathic effect. These results, however, do not resolve whether viral entrance is receptor-mediated or endocytotic, or whether viral replication leads to endothelial cell destruction. Those other factors, such as cytokines, hypoxia, and treatment, contribute to endothelial damage cannot be excluded. Others did not find viral components in endothelial cells by IHC [67, 68] and questioned the nature of the “virus-like particles” identified on electron microscopy [69–72]. Yet, others detected long-term persistence of viral RNA in endothelial cells in the lung [53]. Some data suggest that direct infection of endothelial cells by SARS-CoV-2 occurs and that its effects include hypercoagulation and circulatory disturbances [66]. Although cultures of human endothelial cells could be infected with SARS-CoV-2, the virus did not replicate in the cells and disappeared after 72 h without inducing significant cellular injury [73] that the virus accumulated in endosomes of the endothelial cells and could not be exported into cytosol suggested that it could not replicate [73]. On the other side, senescent endothelial cells were highly susceptible to SARS-CoV-2 infection, causing alterations in gene expression followed by endothelial cell dysfunction, a phenomenon that probably has a role in aggravation of COVID-19 in elderly patients [73].

Our findings add to the discussion of expression of SARS-CoV-2 in the liver in COVID-19. There is no agreement in detection of the virus, even though both hepatocytes and cholangiocytes express the SARS-CoV-2 receptors ACE2 and TMPRSS2. Expression of ACE2 was highest in cholangiocytes, followed by those in sinusoidal endothelial cells and hepatocytes [74–77]. Viral RNA was demonstrated by ISH in Kupffer cells, hepatocytes, and cholangiocytes [8, 13, 30]. A discrepancy also exists between cellular models and detection of virus in vivo and in vitro in hepatocytes [29, 78]. SARS-CoV-2 replication was present in liver organoids [78] and in Huh7 cell lines derived from hepatocellular carcinoma [79]. Whether SARS-CoV-2 can replicate in the hepatocytes, however, is still open [37, 79]. Very recently evidence of virus replication in multiple extrapulmonary tissues was provided early, in the first week following the onset of clinical symptoms; that residual blood persisted within the extrapulmonary tissues was ruled out [8].

The severe acute liver alterations, especially endothelial damage (whether primary or secondary to viral infection), suggest that those patients who survive more severe COVID-19 face a prolonged liver regeneration process, and should be followed accordingly during recovery, with regular checking of hepatobiliary-injury biomarker values even several months after infection. Severe endothelial alterations might occasion microvascular disturbances with long-term complications, which should be taken into consideration in the management of post-COVID patients, especially with co-morbidities affecting the vascular system, such as diabetes, hypertension, obesity, and pre-existent chronic liver diseases.
